# “You really need a whole community”: a qualitative study of mothers’ need for and experiences with childcare support during cancer treatment and recovery

**DOI:** 10.1007/s00520-022-07399-3

**Published:** 2022-10-13

**Authors:** Cheryl Pritlove, Lisa V. Dias

**Affiliations:** 1 Applied Health Research Centre, St. Michael’s Hospital, 30 Bond Street, Toronto, ON M5B 1W8 Canada; 2 Dalla Lana School of Public Health, The University of Toronto, 155 College St, Toronto, ON M5T 3M7 Canada

**Keywords:** Cancer, Parental cancer, Gender, Childcare, Psychosocial care, Qualitative research

## Abstract

**Purpose:**

A cancer diagnosis poses unique challenges for moms with young children who must balance illness-management alongside existing paid (e.g., employment) and unpaid (e.g., domestic/caregiving) work. The goal of this study was to improve understanding of the support needs of mothers living with cancer and their experiences receiving psychosocial and childcare support from a community organization, the Nanny Angel Network (NAN).

**Methods:**

Mothers who accessed NAN services during their cancer treatment and/or recovery (*N* = 20) participated in qualitative semi-structured interviews. Thematic analysis was used to inductively and deductively identify emerging patterns in the data and theoretical abduction was applied to further interpret participants’ accounts using a feminist political economy framework.

**Results:**

Participants expressed how balancing the demands of patienthood and parenthood was challenging and how cancer treatment created new needs for support with care work. Mothers explained that NAN offered indispensable family-centered support largely missing from the health care system, promoting improved physical, psychosocial, and relational health for them and their families. While accessible from a cost-perspective, participants identified different pathways, including awareness, cross-system collaboration, and stable funding, that limited timely access to NAN.

**Conclusion:**

Access to family-centered care, such as that offered through NAN, was vital to the health and healing of the study participants and their families. Improved collaboration with and investment in community organizations like NAN that have a strong infrastructure to support moms living with cancer offers a practical, feasible, and immediate solution to help address some of the distinct challenges this population faces.

## Background

The work of patienthood often involves the adoption of new and added tasks and responsibilities, carried out across changing and often unfamiliar contexts [1, 2]. This constitutes work that is purposeful and requires time, energy, action, and skill [3–5]. Following a cancer diagnosis, patients are tasked with managing the additional labor associated with such things as scheduling and attending medical appointments as well as the management of complex symptoms within the home [1, 2, 6–8]. This is work that can, and frequently does, conflict with existing paid (i.e., employment) and unpaid labor (i.e., domestic and care work in the home) [1, 7, 9, 10]. The work associated with managing an illness like cancer can thus be particularly challenging for parents with young children, who are additionally tasked with balancing their own care with the demands of the family [11–13].

In Canada, approximately 30% of newly diagnosed cancer patients are between the ages of 20–59, an age range that aligns with childbearing and child-rearing years [14]. When confronted with a cancer diagnosis, parents with young children may experience elevated distress associated with the difficulty of assuming multiple roles and responsibilities while ill, an inability to engage in parenting activities as they once had, fear that they will not live to see their children grow up, as well as feelings of anger about the untimely nature of their diagnosis and the things they have lost or missed out on as a result [13, 15, 16]. Endeavoring to maintain a sense of routine and normalcy for their children, many parents feel obligated to continue with tasks, like childcare and housekeeping, despite changes in their own health status and the onset of debilitating side-effects that can render the upkeep of this work challenging [17]. Juggling their own care needs with the demands of the family as a parent living with cancer can be exhausting and lead to burnout [18].

Because women remain disproportionately engaged in the work of social reproduction (maintenance of life on a daily basis, including childcare and domestic chores) [19, 20], the addition of illness-management work can be particularly difficult for mothers with cancer [21–23]. Moms with cancer often find it particularly challenging to attend to their health needs alongside their many obligations to others and are more likely to have stronger feelings of sadness, anger, guilt, and psychological distress while undergoing cancer treatment as compared to other cancer patients [12, 13, 24–27]. Moms of young children also confront a series of practical challenges, such as making the necessary childcare arrangements to attend medical appointments, recover from surgery, and cope with treatment-related side-effects (e.g., fatigue) [28, 29]. Indeed, one study found that over 50% of mothers recently diagnosed with cancer considered childcare to be their most overwhelming responsibility, and that childcare obligations sometimes led to missed medical appointments, and limited their capacity to effectively manage treatment-related side-effects within the home [29]. This can be challenging for all moms, for a variety of reasons, but can be particularly problematic for those who lack the necessary social and/or material resources to secure dependable care for their children [7, 10, 27, 30–32]. In these cases, mothers may “choose” less aggressive, but also potentially less effective treatments, in the interest of attending to more immediate responsibilities, like childcare, with consequences for their own long-term health and recovery [21].

Given the unique circumstances, challenges, and burdens faced by cancer patients who are caring for young children, these individuals may require additional psychosocial services and practical care supports to facilitate improved individual and familial coping and adjustment [15]. The need for tailored and life-stage appropriate programs for parents living with cancer, such as childcare support, has been well documented [12, 33–37]. Despite an expressed need for the development and implementation of social and practical care delivery for this population, there remains a dearth of scholarship pertaining to parents’ experiences accessing such supports and an evaluation of their impacts. Indeed, to the best of our knowledge, there are no qualitative studies that have assessed the impact of childcare supports for mothers of young children who are living with cancer. Identifying and exploring solutions to the practical challenges that parents face when confronted with a cancer diagnosis, like childcare, may be beneficial for patients, their families, and the health care system as a whole [18], thus underscoring the importance and urgency of such investigations. Endeavoring to bridge this gap in understanding, this study provides a gender-informed analysis of the unique work demands placed on moms living with cancer and how these broader circumstances frame their need for and experiences with an organization, the Nanny Angel Network, that delivers life-stage and gender-sensitive psychosocial care, with a focus on childcare supports.

## Methods

### The organization: the Nanny Angel Network

With awareness of the unique and often overlooked challenges that mothers with young children experience during a cancer diagnosis, the Nanny Angel Network (NAN) offers psychosocial and practical care supports aimed at reducing the burden on mothers living with cancer. NAN is a community-based charitable organization that has been operating in the Greater Toronto Area (GTA), in Ontario, Canada, since 2009. The organization provides free services to families experiencing cancer, primarily focused on the delivery of expert childcare services by trained “Nanny Angel” volunteers. NAN services are offered specifically to mothers during periods of treatment, recovery, and palliative care as well as to families during times of bereavement [29]. Nanny Angels are trained in safety and grief support, and coached by Child Life Specialists who practice a modality of care that addresses the social, emotional, and developmental needs of children during traumatic and life-altering events [38, 39]. Nanny Angels arrive at the family’s home prepared with activities, books, and toys to engage children according to their age, needs, and interests. In addition to childcare support, NAN offers mothers access to peer-support groups, a weekly camp-in-a-box program engaging children in virtual activities, and a homework club that provides educational support to children. Further, during the COVID-19 pandemic when provision of in-home childcare was restricted due to necessary public health measures, NAN adjusted their services (e.g., virtual check-ins with moms and children) and provided a meal support program delivering prepared meals to families several times a week. The array of services that NAN offers aims to address the multiple needs of mothers living with cancer, including the need for emotional support (e.g., peer support groups) and support with care work (e.g., childcare and meal preparation). The NAN program further aims to support children, providing age-appropriate information and skills/tools to facilitate coping with a parental cancer diagnosis.

### Theoretical framework

In qualitative research, theory may enter a study at various points throughout the research process, from providing the underlying rationale for a study to assisting with data interpretation [40]. In this study, we took an inductive approach that left us open to the possibility of theory earning its way into the analysis [40]. Because of the frequency of codes pertaining to notions of “family care,” “domestic work,” “self-care,” and “illness-management,” along with the complex, intersecting, and sometimes conflicting nature of discussions contained within these codes, we were oriented toward feminist political economy (FPE) theory when interpreting our findings. FPE provides an analytic framework that theorizes “work” in a broader sense, including unwaged (e.g., volunteer) and precarious labor as well as other forms of work that typically exist outside of (but contribute greatly to) the formal economy, such as the work of social reproduction—work that is disproportionately assumed by women and often invisible within health systems [7, 19, 41]. From this perspective, we were interested in how our participant’s engagement in various forms of work shaped their care experiences and needs, the extent to which NAN was able to facilitate the work of moms with cancer, and the impact this had on their physical and psychosocial health.

### Study design

Our study used qualitative semi-structured interview methods to explore the experiences of moms diagnosed with cancer who accessed childcare support through NAN during their treatment and/or recovery. The Research Ethics Board at St. Michael’s Hospital reviewed and approved the study (REB #19–322). All study participants provided informed verbal consent prior to participating in a phone interview. The verbal informed consent was audio recorded and transcribed verbatim.

#### Recruitment and sampling

Given the study’s interest in better understanding the experiences of moms living with cancer, their need for psychosocial and childcare support, the implications when this support is unavailable, and the impact when such care is provided—NAN offered an apt program through which to achieve our study objectives. Program staff at NAN sent a mass email to their membership, containing a detailed study information letter and contact information for members of the research team. Those who were interested in learning more about the study and/or participating were directed to contact the study coordinator at St. Michael’s Hospital. The study coordinator answered study-related questions, screened individuals to determine eligibility, and scheduled interviews with eligible participants who were interested in taking part in the study. Criteria for participating required that moms: (i) received services from NAN within the past 12 months; (ii) were 18 years of age or older; and (iii) were English speaking. Efforts were also taken to ensure diversity within the sample according to demographic characteristics of age, ethnicity, socio-economic status, and cancer type.

#### Data collection

Recruitment for this study began in late March 2020 when emergency orders were in place due to the COVID-19 pandemic; therefore, all interviews occurred remotely (i.e., over the phone) to protect study participants and research personnel. Interviews were conducted by two experienced qualitative researchers (L.D. and C.P.) between March 2020 and August 2020. The interview guide was designed to better understand participants’ need for support during cancer treatment and recovery as well as their experiences with the NAN program and the services it provides. Probing questions aimed to capture participants’ views of the perceived impact of this program on their physical, psychosocial, and relational health.

#### Data analysis

Interviews lasted between 45 and 105 min in length, with the average interview lasting approximately 75 min. Interviews were audio recorded and transcribed by a professional transcriptionist who also de-identified the transcripts (e.g., removed names, places, or details that could be used to identify the individual). Interview transcripts were uploaded to NVivo 12 software to facilitate data management during phases of in-depth data analysis. Analysis entailed multiple readings of transcripts that included the process of coding data, where researchers assigned a label of meaning (i.e., code) [42] to sections of the interview transcript. The authors (L.D. and C.P.) developed an initial coding framework by reading and analyzing the same four transcripts. This coding framework was then applied to and refined as initial analysis of the remaining transcripts took place. This process resulted in the development of a codebook, consisting of code names, definitions, example data, and analytical summaries [43–45]. The codebook was applied in a second round of coding to ensure consistency of analysis across the interviews. Our primary approach to analysis was thematic analysis, which focused on identifying and naming emerging patterns in the data [46] and included both inductive [47] and deductive [48] interpretations. Following this process, a constant comparative approach was taken to identify and explore similarities and differences within and between interview transcripts [49]. In a final stage of analysis, we employed theoretical abduction [50, 51] to provide further interpretative analysis of participants’ accounts using the conceptual framework provided by FPE.

## Results

### Participant characteristics

A total of 20 individuals participated in the study, at which point it was determined that thematic saturation had been reached [52]. As seen in Table 1, the participants varied according to cancer type, age, education, and employment status. Census data for the province of Ontario, where the participants resided at the time of the interview, reported a median total household income of $74, 287 (Statistics Canada, 2016) and just over half of the participants (55%) reported a household income (before tax) below this median. All the participants lived with at least one dependent child, with a range of 1 to 4 children per study participant. Additionally, most (80%) lived in their home with their spouse or partner and 20% identified as either single, alone, or separated/divorced. Because NAN provides services to a relatively small population of moms living with cancer within a limited geographical area, we opted not to collect and report demographic data on race, ethnicity, and immigration to help protect participant anonymity. Rather, we allowed these conversations to emerge organically in the interview, in a participant-driven way. We report on the ways in which these social identities framed experience and need throughout the “Results” section.

**Table 1:** Demographic and clinical characteristics of participants

Demographic and clinical characteristics	# of participants	Total % of participants (*n* = 20)
Age		
30–34	2	10%
35–39	7	35%
40–44	4	20%
45–49	5	25%
50–54	1	5%
55–59	1	5%
Household income (before tax)		
< $15,000	1	5%
$15,000–19,999	–	–
$20,000–29,999	3	15%
$30,000–39,999	1	5%
$40,000–49,999	2	10%
$50,000–59,999	–	–
$60,000–69,999	4	20%
$70,000–79,999	2	10%
$80,000–99,999	1	5%
> $100,000	5	25%
Unknown	1	5%
Education (highest level completed)		
High school	1	5%
College diploma	4	20%
Undergraduate degree	4	20%
Graduate degree and greater	11	55%
Employment status at time of interview		
Part-time	1	5%
Self-employed	2	10%
Stay-at-home mother	5	25%
Sick-leave	3	15%
Disability	2	10%
Unemployed	4	20%
Other (i.e., student, volunteer, maternity leave)	3	15%
Relationship status		
Married, spouse, or partner	16	80%
Single or alone	3	15%
Divorced or separated	1	5%
Number of children		
1	6	30%
2	8	40%
3	4	20%
4	2	10%
Type of cancer*	Number of participants	
Brain cancer	1	
Breast cancer	12	
Cervical cancer	1	
Colorectal cancer	1	
Endometrial cancer	1	
Non-Hodgkin lymphoma	2	
Ovarian cancer	1	
Thyroid cancer	2	

*One participant was diagnosed with two types of cancer and this is reflected in the table numbers

All of the study participants shared how cancer diagnosis and treatment impacted them and their family. They described the physical toll (e.g., fatigue and pain) of cancer as well as the consuming nature of illness-management and the ways in which this shaped capacity to participate in other important and often necessary forms of work. While disruptions to employment (paid and unwaged) were discussed, most participants emphasized the challenge of juggling illness-management alongside the work of social reproduction. These challenges sometimes led to conflicts, whereby care sacrifices needed to be made. Against this contextual backdrop, participants emphasized how illness-management work added to their already busy lives, the need to prioritize some forms of work over others and the subsequent need to forego certain aspects of care as a result. Occurring in parallel with these discussions were those pertaining to the role of NAN in facilitating work tasks, the value of this support, and the ways in which it fostered improved physical health and psychosocial adjustment. While perceived as critically important care, systemic barriers and existing funding arrangements were believed to limit overall scope and reach of the program as well as timely access to their services. These conversations are unpacked further in the four themes below:

### Adding illness-management work to already busy lives

Participants described an onslaught of new roles and added responsibilities that emerged as a result of their cancer diagnosis. In these discussions, they emphasized the work necessitated by their illness, including the time and energy needed to coordinate, schedule, and attend consults, surgeries, and treatments, as well as the skills required to effectively manage vast and diverse treatment-related side-effects (including pain and fatigue). P11 alludes to the burden of this work and the resource planning and management it demands:

Treatment of cancer, however, is, well, an experience in and of itself. Treatment is really more where the interruption of your life comes in, the side-effects from the medications, and all the time it takes, ah, the number of hospital visits. In my case, I was bumped in between, maybe four hospitals. So, yeah, the treatment side of things is where you really need a whole community, just to get through it (P11).

In addition to the demands imposed by illness-management (e.g., management of appointments and physical symptoms), some participants also described the work involved in coping with the psychological and emotional fallout of a cancer diagnosis. Many of these participants emphasized the struggle associated with facing uncertain futures, both personally and practically, and the work involved in planning for all possible outcomes. As one woman explained:

My mother passed away from pancreatic cancer. So when I got a cancer, I was more, especially that it was spread, I was more thinking about how long I can live. Because, my son only have me, no other people in Canada. So at that point, I was trying to look for, if I died, who is going to adopt him? (P6).

As P6 alludes to, anxieties sparked by uncertain futures were often wrapped up in participants’ concerns about their child(ren)’s well-being. Efforts to repair their own psychosocial health following diagnosis were thus deeply intertwined with the psychosocial health of their child(ren). In turn, instilling feelings of safety and reestablishing a recognizable normal within the home was deemed important work—an act of repairing their own emotional health as well as that of the family. For many, this meant continuing to assume the majority of domestic tasks, like cooking and cleaning; work that was familiar yet changed by the presence of overwhelming cancer-related fatigue and pain. In addition to the continuation of these tasks, participants also described the introduction of new, unfamiliar, and emotionally charged work, like communicating with children about cancer. For many participants, open communication about cancer was seen as being key to supporting effective adaptation among children and to the goal of reinstating stability in the home. However, this constituted work that few felt prepared for, unclear on when to tell their child(ren), what language they should use, and how much detail they should provide. As P10 explains:

Like, how do I tell them [I have cancer]? Do I even tell them? And, I wasn’t sure how to approach that. So, that was something that was [challenging]. And then, my sister was actually the one that said, ‘Well, maybe, if you do tell them, you should tell them before chemo’ because that’s when your physical appearance, like, you can’t hide it (P10).

In the absence of clear guidance on how to navigate these highly sensitive conversations, some participants worried that talking with their children about their cancer could result in additional psychological trauma and thus, ultimately opted not to engage in these discussions. Among these participants, some also recognized, however, that the decision to remain silent about their cancer carried its own potential risks, particularly as markers of the illness became visible (e.g., hair loss, surgical scars) and children were left to wonder what these changes meant.

While many participants remained active in the domestic sphere throughout their cancer journey, there were moments when this work became exponentially more difficult to perform, particularly during treatment, requiring participants to lean on their spouses more and/or to seek outside help. While sometimes reducing the burden of domestic work, this often introduced the work of care coordination. Indeed, nearly all of the participants described needing to secure childcare from their support networks (usually family and friends) following their diagnosis. While coordinating childcare was challenging for most of the participants, for a variety of reasons, this seemed to be particularly difficult for those who had small or informal support networks (e.g., parents of child’s friends, neighbors, members of faith groups). These moms often found themselves tirelessly organizing care that was precarious and frequently unreliable. In their accounts, they emphasized the time, energy, and proficiency that go into accessing these kinds of care supports:

But, then there was, like, randomly, no routine [in childcare]. Like, I spent quite [some] time, try[ing] to coordinate…I spent quite a time, try[ing] to like, ah, just coordinating, [it felt] very draining. Because, you don’t know whether they answer your phone [call], reply [to] your message, whether they have the time, whether they have the intention, a lot of things, so yeah (P6).

The unreliability inherent in precarious childcare supports made it hard for these participants to plan and follow through with illness-management work.

Importantly, all participants described illness-management work as a new and added responsibility, one that compounded already time constrained and busy lives. Juggling various roles and their associated tasks was challenging for all of the participants, but proved insurmountable for some. This was particularly true for those who lacked the social and material resources required to effectively manage and/or delegate responsibility. This often led to competing priorities between work tasks and the need to establish a “work hierarchy” wherein some care responsibilities needed to be prioritized over others. Participants described efforts to creatively maneuver through the demands of both their patient and parent roles, but care sacrifices often needed to be made, the impacts of which are discussed in the theme below.

### The emergence of a work hierarchy and the impact on health and well-being

All of the participants described times when the volume of work was overwhelming and difficult “decisions” about prioritizing work tasks needed to be made. The order of work prioritization varied across the cancer trajectory and was dependent on the social locations of participants, their access to social and material resources, and the difficulty and/or ease with which work tasks could be done. The need to prioritize some forms of work over others and the subsequent need to forego certain aspects of care (for self and/or others) as a result, ultimately impacted upon the physical, psychosocial, and/or relational health of the participants and their families. Below we describe the emergence of work conflicts, the resultant need for care sacrifices, and their various and diverse impacts.

All of the participants in this study were able to make necessary arrangements to attend medical appointment (surgeries and treatments). While most described drawing upon the support of family, friends, and acquaintances (e.g., neighbors), some participants did not possess adequate social and/or material resources to secure outside help (usually those who immigrated to Canada and did not have an established social network in this country). These participants described access to exceptional cancer center staff who either facilitated connections with community organizations that could help or stepped in directly to provide childcare support so that treatments were not missed. This level of care was highly valued and exceptionally helpful, yet exceedingly rare and recognized as being atypical of a standard approach to cancer care within the hospital. In turn, the participants who received such supports tended to position themselves as fortunate or “lucky,” recognizing that in most instances, when childcare support is unavailable, medical appointments often need to be missed or delayed/rescheduled. As the participant below explains:

I was there for three hours, with the injection, and then, they [took] care of my daughter. Because I didn’t have anyone at home. I had to take my daughter to the hospital, which is actually very unusual, because at the cancer treatment center, kids are not allowed. But my oncologist did that. She’s really, really good (P1).

Whether through the support of family and friends, or a health care team who was willing to go “above and beyond,” the participants in this study were able to effectively resolve work conflicts that can arise between the demands of medical care (e.g., consults, diagnostic tests, treatments) and childcare responsibilities. Work conflicts that emerged within the confines of the home, however, were more frequent and typically more challenging to navigate. Indeed, most participants described times where the work of illness-management needed to be juggled alongside, or backgrounded in the interest of everyday tasks; this sometimes resulted in implications for their physical health and healing. As one participant explains:

Just every day care of the kids. I could barely take care of myself...There were mornings that I couldn’t get them out of the house, because physically, I was so depleted. I had no energy. My head would start spinning. I would be weak...having to go and pick them up after school, doing my day-to-day tasks like groceries, house cleaning, helping them with homework after school, it was a lot for me to do by myself, going through cancer treatment, going through chemotherapy, regular trips to the hospital…If I didn’t have the kids, I probably would have been able to manage [cancer] more (P16).

Efforts to maintain a sense of “normalcy” in their everyday lives—usually through continued engagement in the work of social reproduction—was a guiding priority for most of the participants, although this was not always achievable. Indeed, many participants explained that despite their willingness to suspend self-care (e.g., taking time to relax, spend time with their spouse or a friend) and aspects of illness-management (e.g., rest to cope with cancer-related fatigue) in the interest of child/homecare, the physical impact of cancer treatment and treatment-related side-effects disrupted their capacity to engage in the work of social reproduction as they once had. Many participants worried about how their cancer and changed presence in the home was affecting their children. Even among those who had abundant and reliable support networks, there remained concerns about the quality of care. As P11 shared, while family and friends were available to offer support, they were focused on supporting her by attending to practical childcare tasks, like transportation to and from school, but were not as engaged in child-driven care and play:

Everyone else was doing what needed to be done to support ME [emphasis in original]. So, taking her to school, picking her up from school was about helping ME so I wouldn’t have to do it. But, it wasn’t that they were going to pick her up from school and then come hang with her. You know what I mean? They all had their kids too. So, it was just ah, like, that was the one thing; she was surrounded by all these adults, and none of the adults was really there to just hang out with her, just, you know, allow her to be a kid (P11).

For those mothers without reliable or consistent support networks, quality was important; however, the crux of their concerns tended to revolve around the precarity of care and the resultant impact on meeting (or not meeting) the practical needs of the child(ren). As the participant below explains:

Just [to] carry on my daily life, it was very challenging. Like, you know, you drop off [child], pick up [child], if, if I can find people, help me, then they help; like, it’s lucky. If I couldn’t [get childcare support], there are some days, I just didn’t send my son to school…I couldn’t handle…Sometimes, I feel so tired [to] even order some food, and my son wouldn’t like to eat, and I just say ‘I don’t have energy. Whatever the food, you hungry, you eat. Otherwise, you don’t eat’ (P6).

When participants had limited or precarious access to support networks who could provide childcare and/or engage in other forms of domestic work, illness-management often needed to be backgrounded in order to attend to the daily tasks of living in an immediate and sustainable way. As P6 explains above, however, there were times when participants’ bodies were pushed too hard and physical suffering and fatigue rendered the work of social reproduction impossible to perform, with implications for meeting the practical needs of their child(ren).

Prioritizing their own care needs often negatively impacted upon participants’ self-concept as a mother and sometimes contributed to worsened mental health during times when they were simultaneously coping with the psychological and emotional fallout of their diagnosis. This was true for all of the participants, regardless of their access to support networks, many of whom described feelings of guilt for being unable to provide the kind of care they wanted for their child(ren), particularly during a time of heightened need. Referring to this specifically as “mother’s guilt,” one participant explained:

I want to have the best for my kids. And, I feel guilty all the time, that they’re not getting the best of me, that cancer got the best of me (P2).

Another woman similarly remarked:

It has affected me [psychologically/emotionally]. I’m not in my son’s life like I should [be]. I’m not in my husband’s life; even this little one, even this little child I give birth to, I, I’m not really there for her (P7).

The weight of guilt and emotional distress appeared to be particularly prevalent in the experiences of those who noticed heightened feelings of stress, fear, and anxiety in their child(ren). In a broader conversation about the ways cancer disrupts routine and stability in the home, the participants below describe the emotional and behavioral changes they witnessed in their children as a result:

Some people [who provided childcare/transportation to and from school for my son], like, they’re very random. My son, actually [became] very scared to see different stranger[s] face[s]. He was very scared (P6).

I’ve noticed my son has become rebellious. He has become rebellious and, hmm, stubborn. Yes. Before this, before this diagnosis, and this whole cancer journey, my child was, ah, a sweet, soft guy. Right? (Now), he will do, my son will scream back at Mommy (P7).

Efforts to tow-the-line between expectations of patienthood (to focus on illness-management and recovery) and motherhood (to put the care needs of others before your own) were often overwhelming and rife with conflict, demanding participants to make “care sacrifices” in ways that they believed compromised their personal health as well as the health and well-being of their family (particularly their child/children). The burden of work and associated challenge of balancing work conflicts provided the catalyst for outreach to NAN.

### “A pillar” of support: NAN’s impact on mothers’ experiences of care work, health, and well-being

The participants in this study recounted their struggles to effectively juggle various, diverse, and often competing forms of work. At the nexus of work conflicts existed a fundamental tension between the role of patient and that of mom. Despite varying degrees of access to support from social networks, nearly all of the participants described instances where they or their family suffered the consequences of this tension. Yet, the unique challenges that mothers of young children confront when diagnosed with cancer, and the cascading effects of this on the economy of the home, were described as being almost entirely absent from a health care system perspective. NAN, an organization focused on supporting moms with cancer through the delivery of free childcare support, was thus often described as filling an important gap in care.

So, [access to the Nanny Angel Network] that really helps healing. You know, that’s what I see. So, I even mentioned that, Nanny Angel, those kind of people, should be covered by OHIP [The Ontario Health Insurance Plan]. Because it’s really, that’s [what] makes the patient difference. Like, the patient getting better (P6).

During times when participants’ physical and emotional health were compromised, their futures uncertain, and their relationships unfamiliar and sometimes unsteady, NAN was praised for the way it offered stability, with one participant likening her Nanny Angel to a “pillar”—a fundamental component of structural support. Nanny Angels were described as being attuned to and knowledgeable about the unique challenges and needs of a family, particularly children, living with parental cancer. They were touted for their preparation, effective communication as well as engagement in age-appropriate activities and play. In doing so, a number of participants commented on the ways in which Nanny Angel’s helped to restore a sense of normalcy, structure, and security within the home. As the participants below explain:

With the Nanny Angels, obviously, you can feel the love and the care [they had] for my kids, that they wanted to be there, but, there was still structure. They were always teaching my children something, for whatever age group my four children were. Whether it was, even, even down to manners, to sharing, to patience, and even playing one-on-one with them, taking turns, and like, they were playing either board games or they were teaching them things (P14).

So, just engaging my children in doing schoolwork, helping them do their homework, engaging them in activities and taking them out socially. They went tobogganing when I was unable to take them. Ice skating, arts and crafts, science experiments, just everyday childhood play – Nanny Angels [were] able to do that. I couldn’t do it…the day-to-day activity and play of being a child, that Nanny Angels helped me immensely with (P16).

The participants in this study worked hard to protect their children from the uncertainty and distress imposed by cancer and to reinstate a sense of normalcy and routine. Participants experienced varying degrees of success in achieving this goal; however, the work it entailed often came at a great sacrifice to the management of their own health and healing. At other times, particularly during treatment when markers of illness (e.g., hair loss) became visible and side-effects took over, engagement with this work was often impossible. Access to consistent, reliable, caring, and engaged childcare through the support of a Nanny Angel, someone who is knowledgeable about the familial impact of a cancer diagnosis and child development during this time, gave participants comfort and in doing so, gave them “permission” to redirect their focus to illness-management or self-care, without guilt.

[When the Nanny Angel came, my daughter] wasn’t watching TV. She wasn’t playing video games. She wasn’t, she was interacting with somebody. She was laughing. She was getting out her stress. She was having fun…And so, you know [that] allows me to relax a little bit more, not just, not just sit upstairs, you know, I guess, feeling guilty, right? That, ah, you know, that my poor daughter is playing video games, because I’m too tired. Or I can’t colour with her right now…[Having the Nanny Angel here] makes me happier, right? You know, I always want to see, you always want to hear your kids laugh. And, you always want to see your kids doing stuff that they’re enjoying, which is a big deal. You know? I know [daughter] likes to do arts and crafts and likes to sing and dance, and you know, even currently, I can’t have loud music. And I don’t have the patience, (laugh) to hear a song five million times. But that’s what she likes. So to have somebody that can give that to her, or have the patience, because that’s what she enjoys, it means a lot. You know? And then, you know, I get to rest, and not feel guilty that I’m not doing anything. I guess. You know? There’s always guilt as a mother, right? You want them to be the happiest that they can be (P12).

Like P12, most participants described feelings of guilt when their cancer disrupted the stability of the home and when they were unable to prioritize their child in the ways they wanted. In response, many described times when they jeopardized their own physical health and healing to protect and prioritize their children’s needs. When participants were too physically unwell to do so, their self-concept as a mom was challenged and many described experiencing psychological and emotional distress as a result. The presence of a Nanny Angel, someone who prioritized play and offered opportunities for kids to “get out their stress,” helped to resolve the conflicts that participants described between social reproduction and illness-management work. Indeed, the presence of a Nanny Angel made it so that moms did not have to “choose” between their own care or childcare, nor did they have to suffer the emotional consequences associated with prioritizing time for illness-management work.

Many participants shared how having skilled and consistent childcare from a Nanny Angel improved their capacity to manage their illness, such as focusing on rest and attending medical appointments with improved ease. The presence of a Nanny Angel also allowed time for moms to engage in self-care activities like reading a book, socializing with a friend, or spending meaningful time with a spouse, which helped to facilitate improved psychosocial and relational health. Other participants shared how the Nanny Angel’s presence allowed them time to focus on other work in the home, such as preparing meals and/or cleaning, which for some, helped restore their self-concept as a mom, connecting them to a highly valued pre-cancer identity. Below, P3 describes the impact the Nanny Angel had on her ability to rest, recover, and eventually return to everyday tasks:

And, this lady [Nanny Angel] changed (voice breaks) everything in my life, because, at least, on Saturday, I can just sleep-in when she comes. And, they [child and Nanny Angel] go out and they play and they do games in the house. They do art. […] And, she comes for about three to four hours, every Saturday. And, so that became like, my lifeline (voice breaks) and, and helped a lot. And then, as I get better, I started seeing, okay, I could leave her and go get some groceries, at the time. And then come back (P3).

The presence of a Nanny Angel allowed participants to prioritize health and healing in a way that not only fostered recovery but also permitted opportunities to continue with domestic tasks in meaningful ways—facilitating a gradual return to a more recognizable normal.

Beyond the provision of childcare support, NAN also offered support with practical tasks in the home that helped to ease the overall burden of participants’ work. For instance, in response to the risks and restrictions imposed by the COVID-19 pandemic, NAN offered a meal support program and all of the participants who received it expressed great appreciation for the practical support of having prepared food delivered to their homes. Prepared meals reduced the amount of labor they had to do as they spent less time shopping for groceries, planning and cooking meals, and cleaning after meals. Additionally, as one participant highlighted, this service also provided some financial relief to the household:

The food program was unbelievably helpful. Because, being at home, with, you know, five people, and my husband is still expected to work, and [me] having to feed everyone. Aside from the financial assistance, which was a big financial assistance not having to buy food all the time, but not having to worry about every meal, and like, [not] having to literally be in the kitchen the entire day, cooking for everyone, was a very, very, very helpful support (P17).

Another participant explained how the meal delivery service freed-up time, offering opportunities to reconnect as a family or focus on treatment and recovery: “But just, the meals, I don’t have to worry about cooking. My husband doesn’t have to worry about it. We can focus on either family time or treatment” (P12).

In addition to reducing the overall volume and burden of work, NAN also supported moms in navigating unfamiliar and emotionally charged work, including communication with children about cancer. While some participants were comfortable having these conversations and felt they had the skills to do so, others did not. Below P13 alludes to the difficulty of communicating about something as serious as cancer with a child, and describes how a Child Life Specialist at NAN facilitated this conversation in ways that fostered improved coping:

Every time that [I] want(ed) to open the conversation [with my daughter], I just couldn’t talk. I talked to the [Child Life Specialist] and she agreed to help me in that regard. She talked to my daughter about my problem, in a very scientific and psychological way that I really liked it. And, my daughter’s reaction was very good (P13).

Communication with children about the burdens, fears, and uncertainties that accompany a cancer diagnosis was new and unfamiliar work for the participants in this study. Mothers appreciated that Child Life Specialists and Nanny Angels were trained to deal with the psychological and emotional toll that parental cancer can have on children and that they could help facilitate conversations about cancer with children if needed.

Additional services offered through the Nanny Angel Network, such as support groups, further facilitated and encouraged opportunities for health and healing. For instance, the psychosocial impacts of cancer were vast and diverse and were often inextricably linked to concerns about child well-being, yet the participants had few outlets to discuss their feelings, particularly with those who had shared experiences of raising young children while living with cancer. This rendered the work of coping with and rebuilding their psychological health challenging. Participants explained the ways in which a NAN-led support group of young moms with cancer fostered mutual care and a sense of belonging, facilitating improved coping and adjustment, and easing the emotional burden sparked by her cancer diagnosis: “Because, you know, you just, you feel like there are people that care. You’re not alone. And I think that, that makes such an incredible difference, and has made my journey, just every day, a positive one” (P19).

In short, the provision of high-quality and reliable care from an organization designed specifically to meet the needs of moms and families living with cancer was valued by, and valuable to, all of the participants in this study. The personalization and tailoring of services around their unique needs facilitated the type of patient/family-centered care that was missing, and was thus believed to fill an important gap in cancer care delivery.

### Systemic barriers limit the scope, reach, and timely access to NAN services

Participants emphasized the importance of NAN’s commitment to delivering their services free of charge. This was beneficial for all of the participants and facilitated access to some for whom fees would have imposed an insurmountable barrier. Despite being accessible from a cost perspective, the participants in this study described various other pathways that limited timely access to NAN, and posed potential challenges to the scope and reach of the program. These conversations tended to revolve around a general lack of awareness, poor care integration between hospital and community, and lack stable funding arrangements.

While some participants described exceptional social workers or Nurse Navigators who not only informed them about NAN, but facilitated initial contact with the organization, many others described “stumbling upon” the organization. Indeed, most described learning about NAN either by word-of-mouth, pamphlets included in large informational packages, or by staff at other community-based cancer organization (e.g., Gilda’s Club). As P13 describes below, there is no systemic approach to informing patients about this organization, with access relying entirely on the personal practices of individual service providers:

I became familiar with [NAN] as a result of talking with the social worker. If I didn’t talk with [the] social worker, I might not have been able to access [services], because there wasn’t any information about these services at, like, [the] hospital or from doctors or nurses. If they just extend that advertisement for moms with cancer, that would be really good (P13).

Many participants in our study expressed concern about the lack of a systematic approach to awareness raising about NAN services within the hospital, with some explaining that they did not find this resource until late in the course of their treatment or after treatment had ended, and wondered why such important information was not provided from the beginning. They explained that earlier access could have helped to alleviate the high burden of work experienced during treatment, facilitated improved opportunities for illness-management, as well as psychological and relational healing for them and their families. Some participants remarked that there should be a more concerted systemic effort to increase the coordination of this information and to ensure more consistent referral procedures. Reflecting on her own experiences of delayed access to NAN, P19 describes the value of being connected with such an organization at the moment of diagnosis:

I can only imagine what a difference it would make, having that pamphlet [NAN brochure] the same day when you’re diagnosed. Because in that moment, it would give you hope. Like reading everything that they’re doing, and it’s not even more for yourself, it’s more for your kids, because you’re so concerned how they’re going to take it. I feel like that in itself would make your psyche, like, I feel like you would just be stronger, not only for yourself, but for your kids. I feel like you would have a sense of hope, a sense of encouragement, a sense of things are going to be okay, as opposed to feeling unknown, unsure, desperate or you know, in despair, feeling depressed (P19).

With increased awareness and systematic referral processes in place, participants felt that far more moms would seek out services from NAN. However, a number of participants also remarked that current funding arrangements (e.g., private donations) impose significant barriers to the scope and reach of care that NAN could otherwise provide. Indeed, some explained that the lack of adequate and stable funding not only made it challenging to accommodate the volume of patients who could benefit from NAN, but also led to restrictions on program use for existing members, including a 4-h weekly limit on Nanny Angel access per family. While participants understood the need to impose these restrictions, given the vast need for these services, the soft funding under which NAN operates, and the voluntary nature of the Nanny Angel role, most felt they would have benefited from having additional time per week with their Nanny Angel. As P14 explains below:

And it was only once a week, and if I could say too, yeah, four hours is not enough. Like, you want them, like, four hours was fine, but maybe twice a week or something like that. But, I get it, it’s their own volunteer time. So, but yeah, I’d love to have had her [Nanny Angel] over longer (P14).

Having additional Nanny Angel time was discussed as being particularly beneficial during treatment when women’s capacity to engage in domestic work was compromised by cancer-related side-effects, and when child fears tended to be heightened given the presence of physical makers of illness and moms’ changed presence in the home. While immensely grateful that these services exist because of private donors, with some even expressing interest in donating themselves, participants’ accounts also emphasized the ways in which limited and unstable funding can hamper the scope and reach of care NAN is able to provide, even when the necessary infrastructure is in place.

## Discussion

The mothers who participated in this study offered rich accounts of their experiences living with cancer as well as the vast changes and challenges they underwent following their diagnosis and subsequent treatment(s). They expressed how cancer impacted all aspects of their lives, including their physical, psychological/emotional, and social/relational health. As participants’ lives were impacted, so too were the lives of their families. Participants’ worked hard to facilitate their own healing while also working tirelessly to mitigate the impact of cancer in the home. Despite great effort, the burden of work was often insurmountable, leaving them to make difficult decisions about work prioritization and the subsequent need to forego certain aspects of care (for self and others) as a result. In line with other studies on parental cancer [12, 13, 16, 18, 25, 26, 29], this study found that parents are faced with intersecting, and often conflicting, responsibilities that can limit opportunities for illness-management and self-care in ways that can jeopardize health and healing during times of illness. Adopting a feminist political economy lens, we were better able to see the gendered nature of work, the ways in which gender frames work prioritizations during times of illness, and how care experiences, supports, and needs change as gender intersects with other social locations (e.g., income, immigration). Informed by this view, we were able to more clearly and critically evaluate the need for and impact of childcare, and associated supports, received through the Nanny Angel Network.

The findings of this study reinforce and extend existing scholarship on cancer and work, adopting a broadened conceptualization of work [1, 7, 53] to more deeply explore the experiences and challenges of moms who are living with cancer while caring for young children. The participants in this study described an onslaught of new roles and added responsibilities that emerged as a result of their cancer diagnosis. This work needed to be juggled alongside previous responsibilities, most of which related to social reproduction (including childcare, grocery shopping, laundry, and house cleaning) and were tied closely to their role as a mother. Illness-management work was not only added to existing responsibilities, it intersected with them, fundamentally changing the nature of parenting work (e.g., communicating with children about cancer), and the extent to which this work could be reasonably accomplished (e.g., amidst cancer-related side-effects like pain and fatigue). Despite the many challenges, participants nonetheless described working hard to effectively juggle their own care with the myriad demands of family/child care. In these accounts, it became clear how the roles and expectations placed on patients are vastly different than those placed on parents, with the former requiring a degree of “selfishness” (prioritization of self/self-recovery) that conflicts with the selflessness (prioritization of others) demanded of the latter. This conflict may be particularly challenging for women to overcome, as gender norms of womanhood further reinforce a selfless narrative [7, 10]. This provides important context into why many participants in this study (even those with support) continued their engagement in the work of social reproduction, despite consequences to their physical health and recovery. Further, it provides insight into why participants’ psychosocial well-being and relational health within the home were challenged when physical symptoms and side-effects demanded their sustained engagement in illness-management work. From this perspective, we are better able to see the ways in which gender intersects with work to impose constraints on women’s opportunities for balanced (e.g., physical, psychosocial, and relational) care during times of illness. Recognition of the vast social and material resources needed to effectively engage with work tasks, as highlighted in this study, further illuminates key pathways through which inequities between women emerge.

The expectation that people can and will prioritize illness-management work when confronted with a cancer diagnosis, and assumptions that everyone is sufficiently resourced to do so, is particularly disconnected from women’s gendered, political, economic, and socio-cultural lives [7]. And yet, such expectations and assumptions were described as being prominent in cancer care settings, with a number of participants in this study explaining that the unique challenges that mothers of young children confront when diagnosed with cancer, and the cascading effects of this on the home/family, were almost entirely absent from a health care system perspective. NAN, an organization focused on supporting moms with cancer through the delivery of free childcare and other psychosocial (e.g., support groups for moms with young children) and practical (e.g., meal delivery) supports, was thus often described as filling a critical gap in care. What made NAN particularly unique was their recognition that patient-centered care for moms of young children, often meant a family-centered approach to care delivery was needed. The delivery of high-quality childcare services by Nanny Angels who understand the unique challenges and psychosocial needs of children experiencing parental cancer not only helped to reduce the burden of participants’ work, but also gave them “peace of mind,” opening opportunities to focus on their own health and healing without feelings of guilt. Indeed, the delivery of childcare paired with practical services and peer-supports allowed participants to more effectively tow-the-line between expectations of patienthood and motherhood, in ways that facilitated improved health and well-being for them and their families (particularly their child/children). This was true across the participants, regardless of their access to personal support networks, illuminating the difference between child minding services and childcare delivered by trained professionals with expertise in cancer and child development. This finding, paired with what is known about the increased risks of anxiety and stress-related disorders in children of cancer patients [27, 36, 54, 55], adds complexity to the argument that a hierarchy of need for such services should be established [21, 29].

One of the things that made NAN particularly valuable was that it delivered care in the home. For a multitude of reasons, including advancements in cancer care and the subsequent shift of cancer from an acute to chronic condition, the management of this illness is increasingly occurring within the home [56]. While potentially providing a more comforting space for some [57], this shift can also contribute to the development of unique challenges for women. Indeed, the findings of our study support those of others who have found that the home is a site of work for most women, rather than a recuperative space [19, 58–60], with implications for women’s health and healing when they become ill [7, 9]. In turn, while the findings of this study support the value of in-hospital childcare [18, 21], it also illuminates the many limitations of this approach in supporting sustained health and healing for moms living with cancer. Because the relegation of care to the home will likely increase in coming years [56, 61–63], ensuring adequate and timely access to care supports in the community needs to be a priority. This will require improved collaboration between hospitals and community settings as well as sustained financial support for the delivery of community-based care, like the NAN.

There are limitations to this study that warrant consideration in review of the study findings. Firstly, given our interest in better understanding experiences of accessing childcare supports and their perceived impact on moms living with cancer, this study only included the perspectives of those who had access to NAN and were actively receiving childcare services. The experiences and needs of moms who chose not to, or are unable to, access these supports are not captured. Further, while an open invitation to participate in the research went out to all members of NAN (who were currently or had in the past year received services from the organization), participants self-selected into the study and it may be that those who had particularly positive experiences were more likely to participate—potentially as an act of reciprocity for the services they received. While many participants in our study were employed prior to their diagnosis, they were on leave at the time of the interview and thus not assuming the additional labor associated with paid work. Given our finding that care sacrifices often needed to be made when work conflicts could not be managed, an improved understanding of the experiences and needs of moms who continue to engage in paid employment during treatment is warranted. Lastly, while the mothers in our study shared their perceptions about the impact of NAN services on the psychosocial adjustment and well-being of their children, the perspectives of children themselves are not captured. Given what is known about the psychosocial impact of parental cancer on children [36, 55], an evaluation of children’s experiences with and perspectives about interventions designed to support them, like the NAN, might help to improve the personalization of service delivery to meet their unique needs and are thus urgently needed.

## Conclusion

The findings of this study illuminate how women’s socio-cultural lives can conflict with their own health and recovery during the upheavals of illness. This study further highlights how the invisibility of this conflict from a health care system perspective has contributed to a general absence of supports to meet the needs of young moms living with cancer. The invisibility of the unique challenges that moms face may also explain the lack of a systematic approach to collaboration between hospitals and community organizations, like NAN, that our participants described. Given the vast and diverse benefits that our participants and their families experienced as a result of their involvement with NAN, we argue that improved collaboration and more stable funding arrangements (e.g., governmental/public funding support) could help to ensure that moms with cancer receive the support they need when they need it. The provision of childcare supports provided through NAN better positioned the participants in our study to engage in illness-management and promoted improved physical, psychological/emotional, and social/relational health for them and their families. From this perspective, timely and sufficient access to person-centered psychosocial and practical care, such as that offered through NAN, could improve illness outcomes, bridge inequities in care, and improve overall quality of life. Making use of, and financially investing in, community care organizations like NAN that have a strong infrastructure to support mothers living with cancer offers a practical, feasible, and immediate solution.
